# Arthropod Vectors and Disease Transmission: Translational Aspects

**DOI:** 10.1371/journal.pntd.0004107

**Published:** 2015-11-19

**Authors:** Wolfgang W. Leitner, Tonu Wali, Randall Kincaid, Adriana Costero-Saint Denis

**Affiliations:** 1 Division on Allergy, Immunology and Transplantation, National Institute of Allergy and Infectious Diseases, National Institutes of Health, Bethesda, Maryland, United States of America; 2 Division of Microbiology and Infectious Diseases, National Institute of Allergy and Infectious Diseases, National Institutes of Health, Bethesda, Maryland, United States of America; University of Perugia, ITALY

## Introduction

Numerous diseases are transmitted by arthropod vectors, and for many of those diseases, effective vaccines are still not available. The contribution of the vector to the process of pathogen transmission is often overlooked, despite providing new avenues for combating vector-borne diseases, some of which could complement and significantly enhance ongoing efforts. To explore novel approaches to fighting vector-borne diseases, the National Institute of Allergy and Infectious Disease (NIAID) convened a workshop with experts in parasite immunology, vector biology, and entomology (listed in Table 1), who discussed possibilities of translating these basic research ideas into potential commercial products. The feasibility of product development was analyzed for four types of approaches: the use of vector-derived factors, such as arthropod saliva, as vaccine candidates to prevent transmission; the evaluation of bioactive vector saliva proteins as novel drugs; the use of vector saliva molecules as biomarkers of vector exposure; and the modification of the vector microbiome to alter vector competence. Some of these approaches are highly promising, some are already quite advanced, and all have the potential to significantly reduce the transmission of vector-borne diseases. However, the discussions also revealed significant regulatory and market challenges in the path toward a commercial product, even for the most promising approaches.

**Table 1:** Names and affiliations of speakers at the NIAID meeting “Arthropod Vectors and Disease Transmission: Translational Aspects” held May 2014.

Speakers	Affiliation	Title of Presentation
Shaden Kamhawi	NIAID, NIH	Development of a *Leishmania* vaccine
Maha Abdelahim	NIAID, NIH	Sand fly saliva as adjuvant
Iliano V. Coutinho-Abreu	University of California, Riverside	Translation of sand fly saliva to a vaccine
Jose Ribeiro	NIAID, NIH	From bugs to drugs—a translation story
Anne Poinsignon	Institut de recherche pour le développement (IRD), Montpellier, France	Salivary factors to activate *P*. *vivax* hypnozoites
Joao Pedra	University of Maryland	Tick saliva and NLR signaling: A potential for therapeutics
Frank Remoue	Centre de Recherche Entomologique de Cotonou (CREC), Benin	Salivary factors as biomarkers
Andre Sagna	Espoir pour la Sante, Sénégal	Salivary factors as biomarkers for infectious bites
Zeljko Radulovic	Texas A&M, USA	Vector factors and immunomodulation: Potential source for immunotherapeutics
George Dimopoulos	Johns Hopkins University, Baltimore	Symbionts: The road to pathogen control
Nathan Dennison	Johns Hopkins University, Baltimore	From mosquito bugs to malaria drugs
Job Lopez	Mississippi State University	Tick saliva: Potential relapsing fever vaccine
Pamela Pennington	Universidad del Valle, Guatemala	Applying paratransgenesis for disease control

## Novel Vaccines That Target the Vector, Not the Pathogen

Traditionally, vaccines against vector-borne infectious diseases target antigens expressed by the infectious agent in an attempt to neutralize the pathogen or, at least, reduce the disease burden and, thus, reduce morbidity and mortality associated with the disease. However, a few vaccines instead target pathogen-associated antigens expressed during life stages of the parasite that are associated with uptake by a vector (e.g., gametocyte) or development inside the vector. Such transmission-blocking vaccines represent a distinct second category of vaccines against vector-borne diseases and provide benefit to the community in an endemic area, not in terms of affecting disease pathogenesis, but by limiting the spread of the pathogen. A separate category of transmission-blocking vaccines (vector-targeting, transmission-blocking vaccines) targets molecules inside the vector, such as the Galectin PpGalec (Table 2) in the midgut of the sand fly [1]. When antibodies in the blood meal of the sand fly’s host bind to these molecules in the vector’s gut, they interfere with the attachment and, thus, development of *Leishmania* parasites. The discovery that vector saliva includes immunosuppressive or immunomodulatory molecules, which facilitate the establishment of an infection (reviewed in [2]), has given rise to a third category of vaccines against vector-borne pathogens. The objective of these vaccines is simple and elegant: Targeting vector saliva molecules that assist pathogens during infection may either “unmask” the infectious inoculum and allow the host’s immune system to eliminate it (e.g., tick-borne diseases as reviewed in [3]), or induce an immune response that interferes with the establishment of an infection by the vector-borne pathogen (as shown for *Leishmania* [4,5]). Such vaccines could, in principle, be effective against multiple infectious diseases transmitted by the same type of vector and would not be rendered ineffective by mutations in immunodominant epitopes on pathogen-derived antigens, which conventional vaccines against infectious diseases target. Despite the identification of numerous potent, immunomodulatory saliva molecules from a broad spectrum of blood-feeding arthropods, the vast majority of studies investigating them as vaccine candidates have, unfortunately, not moved beyond early preclinical studies. Such saliva-based vaccines may have potential as stand-alone products or, more likely, as an adjunctive component of traditional vaccines against vector-borne diseases. In the latter case, they may be able to reduce the infectious inoculum during a blood meal and facilitate recognition of the infectious agent by the vaccine-primed host immune system. Combining saliva-based with pathogen-based vaccines [6], though scientifically highly appealing, is, however, significantly more complicated from a regulatory and intellectual property standpoint, as well as a manufacturing and formulations standpoint, since it would involve multiple active components.

**Table 2:** Accession/ID numbers of proteins mentioned in the article.

Protein	*Species of origin*	Accession number
PpGalec	*Phlebotomus papatasi*	GenBank AAT11557.1
gSG6	*Anopheles stephensi*	GenBank AAO74842
LJM11	*Lutzomyia longipalpis*	GenBank AAS05318.1
SP32	*Phlebotomus papatasi*	GenBank AFY13225.1
Ixolaris	*Ixodes scapularis*	GenBank AAM93647.1

Of all vector saliva-based vaccines, those designed to prevent leishmaniasis are the most advanced. *Leishmania* infection represents an excellent choice for a first-in-class vaccine targeting the vector for the following reasons: an effective human vaccine against leishmaniasis is still not available; it is a widespread, albeit neglected, tropical disease; and the vaccine does not necessarily have to be administered to humans, since it could be used to target other mammalian hosts such as dogs in an effort to reduce transmission from animal hosts to humans. An approved and effective veterinary vaccine would also represent a significant stepping stone for the subsequent development and approval of a human vaccine. The lower regulatory bar for veterinary vaccines, and particularly for animal species not used for meat production, makes the targeting of animal reservoirs—rather than human hosts—an attractive approach.

Research on sand fly saliva as a vaccine against *Leishmania* parasites has yielded valuable insights into the mechanism of protection mediated by vector antigen-based strategies: unexpectedly, protection by such vaccines is mediated not by neutralizing antibodies against the targeted saliva antigens, but by a Th1-biased delayed-type hypersensitivity (DTH) response capable of preventing vector-transmitted leishmaniasis in mice, hamsters, dogs, and primates. In these animal models, the protective immune response induced by the vector saliva-based vaccine broadened after parasite challenge to also include immunity against *Leishmania* antigens, thus representing a unique and highly desirable form of epitope spreading.

While saliva antigen-specific antibody responses were found not to be relevant in the case of *Leishmania* infection, would it still be useful to develop vaccines that primarily induce a humoral response? Anti-saliva vaccines may be used to target saliva proteins essential for blood feeding to reduce the size of the blood meal. In theory, this would interfere with the ability of the vector to obtain a full blood meal, resulting in reduced egg production and, thus, decreasing the vector population. Whether such a strategy would result in vectors seeking more, but smaller, blood meals would need to be addressed carefully, since it may result in an inadvertent increase in disease transmission. This strategy may also be more appropriate for ticks rather than winged and more mobile vectors.

A number of useful insights have been gained in the area of research on vector factor-based vaccines: (1) Despite considerable progress in characterizing vector saliva components, finding the most appropriate vaccine candidate(s) in a complex mixture of saliva proteins is a highly empirical process, especially when the (immunological) function of individual proteins is not known. This slows the development of new vaccines, and represents a challenge for academic researchers to develop appropriate assays to screen candidates for functional responses. (2) The identification of the ideal vaccine platform, as well as a suitable adjuvant, to deliver a vector-derived antigen will require extensive basic research, because it is unknown what type of immune response against the vector antigen is protective. (3) One reason for vaccine failure in field trials against infectious diseases (e.g., malaria) has been the heterogeneity of the target antigen expressed by different strains of the pathogen. It will be important to avoid a similar shortcoming of vector-antigen vaccines by determining the variability of the antigen between vector subspecies found in the area where the vaccine will be deployed. (4) Molecular mimicry is a relatively common phenomenon and represents an immune escape strategy used by various pathogens. The expression of pathogen-derived antigens that resemble host proteins has been linked to autoimmune diseases after exposure to certain pathogens, such as systemic lupus erythematosus (SLE) following malaria infection or Chagas disease following infection with Trypanosomes [7,8]. Proteins in vector saliva, however, have been under little to no evolutionary pressure to avoid the immune system of host species by using molecular mimicry, thus eliminating concerns about their potential to break immunological tolerance to structurally similar host proteins—and induce autoimmunity—when used as vaccine candidates.

## Immune Responses to Saliva Proteins As Biomarkers of Exposure

The vertebrate host mounts an antibody response to at least certain arthropod saliva proteins, providing a “signature” or “record” of prior—and mostly recent—vector exposure, despite the minute amounts of saliva injected into the bite site, and despite the host’s suppressed immune response to the inoculum. It remains unclear if these antibodies significantly affect the ability of the vector species to obtain an efficient blood meal or interfere with a vector-borne infection since certain saliva proteins clearly aid during the initial stages of infection. It is possible that the subtype or specificity of those naturally induced anti-saliva antibodies is significantly different from those induced by a saliva protein-based vaccine. Nevertheless, the bite-induced humoral response represents a useful indicator of bite frequency. Humoral responses against saliva proteins correlate well with the extent of exposure at a population level and can, therefore, be used as an objective method to assess the usefulness of vector-control measures. While the majority of susceptible animals in an endemic area tend to be uninfected, the relative rate of infected vectors can easily be determined and, together with the serological data from those living in the area, be used to estimate the risk of infection.

What are the practical considerations for using vector-specific antibody responses as biomarkers of vector exposure? While the heterogeneity of saliva proteins between vector species limits the broad-based effectiveness of saliva-based vaccines, the uniqueness of a vector species’ sialiome makes it possible to determine what type of vector has been feeding on a host. Therefore, vector-specific test kits are feasible and could be used to estimate the risk of infection from diseases transmitted by a particular vector species. However, research has clearly shown that measuring overall IgG responses against whole saliva of a particular vector species, such as *Anopheles* mosquitoes, may not be a useful approach. In this case, test kits will need to be based on individual and carefully selected saliva proteins and/or specific salivary peptides. An example of such a candidate is gSG6-P1 (Table 2), a peptide from the saliva of *Anopheles*. It is found in all *Anopheles* species, is antigenic (recognized by specific antibodies) in exposed individuals from major endemic areas (Africa, South Asia, South America), is unique to the genus, and does not show cross-reactivity with saliva proteins from other vector species. Exposure markers for sand flies have also recently been identified, namely LJM11 (Table 2) and LJM17 (Table 2) for *Leishmania lutzomyia* [9] and the SP32 (Table 2) protein for *Phlebotomus papatasi* [10,11]. In contrast to these vector species, non-fractionated saliva from *Glossina morsitans submorsitans* may be a useful reagent for assays designed to determine exposure to the vector [12]. Thus, the requirements for such test kits will be highly dependent on the vector species.

The first generation of such vector-exposure kits is already within reach, but they will not address the question of whether or not vector bites had been infectious. Determining the frequency of infected vectors in a particular area requires tedious, manual analysis of captured animals. However, the ratio of infected versus uninfected vectors counted in the field does not necessarily reflect the frequency with which the two vectors bite their hosts. To determine the exposure to infectious bites more reliably and conveniently, it may be possible to take advantage of the fact that infection can change the saliva composition of the vector (e.g., the changes in tsetse fly saliva due to trypanosome infection [13]). Therefore, if immunogenic proteins, which are uniquely associated with the saliva of infected vectors, can be identified, they may be included in second-generation test kits. With such kits, the ratio of antibodies against “constitutive” and infection-induced salivary proteins could be used to quickly and easily determine the relative rate of infectious bites compared to the total number of bites an individual in an endemic area receives. Such measures of vector exposure could be supplemented by the measurement of antibody responses to pathogen-derived antigens, providing a basis for the comprehensive analysis of pathogen transmission. This would provide a powerful and rapid diagnostic tool to evaluate vector control measures, vector infectivity, and seasonal changes in both parameters, as well as allow the geographic identification of infection hotspots for a more targeted deployment of vector control measures. It would also provide a valuable tool to monitor areas declared free of a disease, e.g., malaria, after an elimination campaign to be able to rapidly and effectively respond to any re-emergence of the disease.

## Bugs to Drugs

The saliva of blood-feeding arthropods contains bioactive molecules, which have evolved over the course of more than 100 million years to exert specific pharmacological effects even when only minute amounts are present at the bite site. They modify the bite site to facilitate the blood meal, but are also exploited by vector-borne pathogens, which take advantage of the immunosuppressive environment established by certain saliva proteins. The vector sialome is an enormous, largely unexplored, and virtually untapped source of pharmacological agents. Its size is largely due to the fact that blood feeding was independently invented by multiple vector species, thus giving rise to multiple sets of evolutionarily unrelated saliva proteins with overlapping functions (e.g., anticoagulants, which are used by virtually every vector species).

Vector saliva proteins that can be produced by recombinant expression methods have a variety of potential applications: Saliva components that function to skew immune responses (either to a Th1 or Th2 phenotype) may be useful as immunomodulators, for example, in the treatment of autoimmunity, or as vaccine adjuvants. Several saliva proteins exhibit immunosuppressive activity, such as Sialostatin L2, which suppresses inflammatory responses and could be used for the treatment of inflammatory diseases [14–16]. Salivary glands of sand flies are the source of several anti-inflammatory molecules, such as LJM111 from saliva of members of the genus *Lutzomyia* [17], or nucleosides from the saliva of *Phlebotomus*, which impair dendritic cell functions [18]. Such molecules may find applications in the treatment of arthritis and other inflammatory diseases. Similarly, clinical applications can be envisioned for the many vasodilators, anticoagulants, cytokine modulators, histamine-binding proteins, complement inhibitors, or Ig-binding proteins found in the saliva of blood-feeding arthropods. A specific example cited at the workshop was the treatment of pulmonary arterial hypertension with inhibitors of tissue factor such as *Ixolaris* (Table 2) from ticks [19,20]. This particular molecule has also shown potential as a tumor therapeutic agent and for treating macular degeneration and arthritis. The potential for using vector saliva factors to combat autoimmune disease or as immunosuppressive agents after organ transplantation should also be considered and explored.

What are the potential disadvantages of using saliva protein-based therapeutic agents? Unlike most small molecules, such foreign proteins will eventually trigger the induction of neutralizing antibodies, which will limit the duration of their use in an individual patient. This may be circumvented by taking advantage of the broad variety of evolutionarily and structurally unrelated saliva proteins from different vectors, which have the same or similar biological functions. This approach would mimic the strategy of blood-feeding ticks, which are capable of using multiple gene-loci-encoding saliva proteins with overlapping functions, but which are immunologically not cross-reactive. Therefore, despite the extended exposure of the host to tick saliva proteins, no neutralizing antibodies are induced. Field studies suggest that antibody responses against saliva proteins triggered by mosquito bites are short-lived [21], but it is not known yet whether this phenomenon is related to the nature of these proteins or to the small amounts delivered during a blood meal.

## The Vector Microbiome, or “Bio-prospecting Vectors for Microbes of Interest”

Traditionally, efforts to combat vector-borne diseases have focused on either vector control or the elimination of the pathogen inside the host following transmission through an arthropod vector. However, both approaches have significant limitations. Insecticides can be quite effective in eliminating a vector species temporarily and locally, but this approach is expensive. For example, a nationwide spraying campaign was conducted in Guatemala starting in 2002 to combat triatomine-transmitted Chagas disease [22] at a cost of US$10/house, but the impact of the campaign was short-lived. Long-term application of pesticides harms other species (e.g., honey bees) and results in the selection of insecticide-resistant vectors. Bed nets protect against blood-feeding by vectors during peak times of transmission only when properly used and maintained. Environmental modifications such as the draining of swamps are not feasible everywhere and are extremely costly, in addition to having potentially devastating effects on the environment. Finally, repellants only work as long as they are used properly (i.e., continuously), thus limiting their usefulness.

Similarly, pathogen-directed therapeutic and preventative measures have numerous limitations. Drugs targeting the pathogen eventually and inevitably result in the selection of drug-resistant pathogen strains. They are expensive (particularly for developing countries), and frequently have undesirable side effects. Vaccines are the most attractive of all strategies, based on a cost-benefit calculation and the potential to protect against infection for extended periods of time. However, vaccines against vector-borne diseases have been largely unsuccessful so far due to insufficient immunogenicity and efficacy. Furthermore, immune responses against many pathogen-derived antigens are unexpectedly short-lived. Other reasons for failure in field trials include an insufficient understanding of the targeted antigen. For example, AMA-1 was thought to be an essential protein for the invasion of host cells by apicomplexan parasites such as *Plasmodium* or *Toxoplasma* [23], and became the focus of intense vaccine research. However, subsequent studies demonstrated that the antigen is dispensable for host cell invasion. The sheer complexity of host-pathogen interactions, including manipulation of the host immune response, represents a formidable, though not insurmountable, challenge to design effective vaccination strategies. A valuable lesson learned from trials with the RTS.S malaria vaccine has been that the efficacy of a vaccine against a vector-borne disease could be dramatically improved by simply changing the vaccination regimen [24].

Workshop participants discussed an alternative approach to control vector-borne pathogens, one focusing on a stage of the transmission cycle which has received little attention until recently—the time a pathogen spends inside the vector. The infection of a vector and the infectivity of this vector are, to a significant extent, controlled by the vector’s immune system. This makes the vector’s own immune defense mechanism an attractive target for intervention, since it may be possible to enhance it to a point where the vector can either no longer be successfully infected or can eliminate the pathogen. A major obstacle in developing such strategies is the highly limited understanding of the arthropod immune system. A recommended solution was to enhance integration of research on the immune system of vectors and *Drosophila*, with the latter being significantly more advanced.

What approaches could be used to make vectors pathogen-resistant? Two strategies were discussed: First, it is possible to engineer vectors that constitutively over-express immune defense genes. Unfortunately, the constitutive expression of such genes results in a variety of issues that affect the health and survival of such animals in the field. However, by placing the vector’s immune defense genes under the control of promoters from genes that are activated by blood feeding, the vector’s immune status could be temporarily enhanced following a blood meal that contains the pathogen. As with any genetically-modified species, it is essential that a modification will not affect the animals’ life span, fecundity, or overall fitness. Any negative impact on those parameters, even if very minor, will prevent the modified vectors from replacing the wild type population and potentially doom the success of an expensive release of these organisms. Inevitably, this requirement will raise safety concerns, since the modified population could no longer be controlled after release, which raises the bar for safety studies and increases regulatory scrutiny.

The second strategy is based on replacing the vector’s microbiome with microorganisms that impact the vector’s pathogen load [25]. This approach has already been explored in field trials. Replacement microbiota may represent unmodified microbial species that normally do not colonize a particular vector species, or genetically engineered symbiotic bacteria [26]. Various distinct mechanisms can mediate the inability of the vector to transmit pathogens: (1) The newly introduced microorganism may directly affect the pathogen’s ability to colonize the vector and survive in it (e.g., anti-*Plasmodium* effector proteins produced by *Pantoea agglomerans* [26]). (2) The vector’s immune system may constitutively respond to the presence of the animal’s microbiome, thus ramping up the immune status of the vector (e.g., by *Enterobacter cloacae* [27]). (3) The microorganism may shorten the vector’s lifespan and thus interfere with the transmission of pathogens that require a relatively long period of development in their arthropod host (e.g., *Wolbachia pipientis* wMelPop, which blocks Dengue transmission through this mechanism [28]). The first two phenomena are not restricted to arthropods, but have also been observed in vertebrates, including humans. The potential for blocking disease transmission by altering the vector microbiome has received considerable attention in recent years, following the high-profile releases of vectors carrying modified microbiota; specifically, *Aedes aegypti* mosquitos infected with a mosquito-adapted *Wolbachia* strain obtained from *Drosophila* have been successfully released in Australia and other countries to control dengue transmission. This strategy successfully interferes with the transmission of *Plasmodium* [29] as well as other vector-borne pathogens. Data from large-scale releases indicate that the approach appears to be safe and does not appear to have unintended negative side effects. Since *Wolbachia* is a ubiquitous microorganism, no new organism is being introduced into the environment by this strategy.

A vector’s microbiome can be altered either through the stable “conversion” of vector populations in the wild or by introducing the desirable microbiota through bait stations, which allows for a continuous modification of vector populations. The latter approach is particularly useful for microbiota, which are not (or only poorly) transmitted horizontally, or vertically. The bait has to be cheap and designed for a particular vector species (e.g., CRUZIGARD is specific for *Rhodnius* by simulating feces; *Anopheles* bait offers sugar water or nectar). Alternatively, lab-generated (paratransgenic) colonies of vectors are released with the objective of eventually pushing out the naturally-occurring populations. The latter approach, while technically more attractive, faces significant hurdles in part because the necessary regulatory framework does not yet exist, and because of public resistance when the released vector species is perceived to be a genetically modified organism.

*Wolbachia* is particularly suitable as a “replacement microbiome” since it is efficiently transmitted between mosquitoes and, thus, easily maintained in the vector population after release. However, there are likely many more useful microbial species that simply have not yet been explored. They may be used by themselves or as part of a microbiome cocktail (together with *Wolbachia*) to further improve the effectiveness of this strategy, and as the “replacement microbiome” for additional vector species. The analysis of novel microbiota that prevent the pathogen colonization of vector species may also accelerate the identification of novel therapeutic agents, such as an antifungal cyclic dehydropeptide lactone isolated from *Aeromonas* [30]. This approach to identify novel therapeutics against infectious diseases may be more attractive than the currently used screening of (existing) libraries of chemical compounds for several reasons. First, compounds produced by bacteria with antimicrobial activity have been evolutionarily selected and optimized and, second, a highly relevant and relatively inexpensive in vivo screening system is already available in the form of the vector animal [31,32].

## Product Development: Lessons for the Field of Vector-Based Intervention

The process of “translating” exciting scientific ideas into tangible products begins with a simple acknowledgment—that the motivations and goals of basic scientists differ from those involved in the business of developing products. Numerous non-scientific aspects need to be considered and addressed before advancing a promising scientific discovery, such as those discussed above, into the pathway for product development. Most of these translational considerations are pragmatic ones and require a fundamentally different mindset than that found in basic research, which often seeks to explore and pursue new ideas. By contrast, business development is driven by cycles of planning and risk assessment designed to anticipate known problems that are often encountered by product developers. There are a few basic guidelines that may be helpful to those seeking to go beyond the bench and into the world of translational development.

First, it is important to clearly articulate the properties and anticipated uses of the product—the so-called “target product profile.” This helps focus early discussions on the attributes (intended use, target population, potency, specificity, stability, tolerability, ease of manufacture, etc.) that the product must have to be successful. For example, for a vector saliva-based product, the considerations may include the following: Is the target population humans or an animal host which serves as a reservoir for the pathogen (e.g., dogs in the case of *Leishmania*)? Is the salivary antigen highly variable between vector species from different geography areas? Is it enough to include one protein in the vaccine or should multiple antigens be targeted to increase efficacy and overcome potential variability? What vaccine adjuvant is required to obtain an effective immune response, and has that adjuvant previously been used in humans? Although potentially essential for obtaining adequate immunogenicity, a human vaccine that includes both a novel antigen (*i*.e., vector saliva proteins) and novel adjuvant (i.e., adjuvants not part of a licensed vaccine) will face more regulatory scrutiny. Can the formulated vaccine be stored at 4°C, does it have to be stored frozen, or is bedside mixing of components required? The latter two significantly complicate deployment and delivery.

Second, it is crucial to identify a suitable customer or a collection of stakeholders for the novel product, since the end-users (inhabitants of endemic areas in developing countries, for most of the innovations discussed here) may not be able to afford even reasonably priced products. Therefore, it is necessary to determine at the outset the level of interest by potential benefactors and/or investors (government agencies, non-governmental organizations [NGOs], or companies), a challenging process that requires a significant outreach effort.

Third, a major consideration influencing product feasibility is the regulatory path that will be required. In some cases adequate precedent exists, based on similar products and their intended uses. In the case of saliva-based vaccines, challenges include the fact that there are no licensed (human) vaccines yet that are based on vector saliva, although a veterinary vaccine against tick-borne pathogens (TickGARD) has been on the market for many years. Furthermore, a vector saliva-based vaccine may require an adjuvant that had not previously been used in a vaccine licensed for use in humans. Using technologies already in place can accelerate the process of commercializing a novel approach. For example, for the continuous introduction of novel microbiota, it is advisable to explore the usefulness of already available bait stations. For novel diagnostic kits, it is advisable to determine whether commercially available testing kits could be modified. The benefit of a smoother commercialization pathway is, however, partially offset by the need to enter into licensing agreements with the intellectual-property owners of the platform technology in question. In those cases where a truly novel solution is proposed, substantial early discussion and negotiation with regulators may be required; a case in point might be the introduction of vectors with modified microbiomes for which no regulatory framework exists in most countries.

Finally, most research scientists have limited or no business experience. When taking a new invention to market, it is essential to find a business partner to share the responsibilities of product development. An experienced businessperson will likely bring other issues forward, including competitive advantage over existing products, macroeconomics, marketing strategy, and long-term sustainability of the market. Concepts like these are often viewed as unfamiliar and uninteresting by research scientists, but they may determine if a good scientific idea actually makes good “business sense.” The juxtaposition of these world views requires that scientific investigators relinquish some control over the innovation and not attempt to micromanage the product development process.

Even if one is able to find sponsors for a novel product and overcome the major scientific, technical, and regulatory hurdles, a new product to combat vector-borne diseases may face yet another set of challenges: the cultural and ethical standards of the environment where it will be used and deployed. Even products that are approved by regulatory agencies may not be aligned with the cultural norms required for acceptance of these new approaches, as the experience with genetically modified organisms has shown. It should be expected that novel technologies and approaches will elicit concerns and apprehension in the target population. Outreach, advocacy by local leaders, and education of the intended population very early in the process of deploying the new technology can make the difference between successful engagement and rejection. The issues surrounding product commercialization and sustainment represent a daunting challenge, but not an insurmountable one. What is needed is an appreciation of the careful planning needed to anticipate and overcome key risks and the many factors that contribute to achieving success.

**Key Learning Points**

- Numerous infectious diseases are transmitted by arthropod vectors, and effective vaccines against these diseases are still lacking.
- Vector-derived molecules such as saliva proteins are actively involved in the transmission process and are attractive targets for the development of novel vaccines against vector-borne diseases, since protection cannot be bypassed through mutations in pathogen-associated proteins and since they may prevent infection rather than inducing an immune response against an establishing or established infection.
- Antibody responses to vector saliva proteins are reliable indicators of the exposure to vector bites and can, for example, be used to monitor the effectiveness of vector-control strategies.
- Because of their potent biological activities, vector saliva proteins are being explored as novel pharmaceuticals, for example, as anticoagulants or immunomodulators.
- The microbiome of the vector significantly influences the ability of pathogens to be transmitted by vectors. Replacing, or adding to, a vector population’s microbiome with appropriate microbiota (such as *Wolbachia*) can significantly reduce transmission rates of vector-borne diseases.
